# BEAT^® ^the bispecific challenge: a novel and efficient platform for the expression of bispecific IgGs

**DOI:** 10.1186/1753-6561-7-S6-O9

**Published:** 2013-12-04

**Authors:** Pierre Moretti, Darko Skegro, Romain Ollier, Paul Wassmann, Christel Aebischer, Thibault Laurent, Miriam Schmid-Printz, Roberto Giovannini, Stanislas Blein, Martin Bertschinger

**Affiliations:** 1 Cell Line Development and Protein Expression group, Glenmark Pharmaceuticals SA, La Chaux-de-Fonds, 2300, Switzerland; 2 Antibody Engineering group, Glenmark Pharmaceuticals SA, La Chaux-de-Fonds, 2300, Switzerland; 3 Downstream Processing group, Glenmark Pharmaceuticals SA, La Chaux-de-Fonds, 2300, Switzerland

## Background

The binding of two biological targets with a single IgG-based molecule is thought to be beneficial for clinical efficacy. However the technological challenges for the development of a bispecific platform are numerous. While correct pairing of heterologous heavy and light chains (Hc and Lc) can be achieved by engineering native IgG scaffolds, crucial properties such as thermostability, effector function and low immunogenicity should be maintained [1]. The molecule has to be expressed at industrially relevant levels with a minimum fraction of contaminants and a scalable purification approach is needed to isolate the product from potentially complex mixtures. This article introduces a novel bispecific platform based on the proprietary BEAT^® ^technology (Bispecific Engagement by Antibodies based on the T cell receptor) developed by Glenmark.

## Materials and methods

Stable cell lines were generated by co-transfection of three proprietary expression vectors pGLEX41_GA/GB coding for the Hc, Lc and Fc-scFv under optimized stoichiometric conditions in CHO-S cells. Cell lines were selected according to expression and heterodimerization during small scale fed-batch cultures performed in TubeSpin bioreactors (TPP, Trasadingen, Switzerland). For high throughput (HT) screening, the fraction of BEAT^® ^molecule was evaluated using the Caliper LabChip GXII Protein Assay (PerkinElmer, Waltham, Ma, USA). Titers were measured by HPLC-PA after 14 days of culture. The fraction of heterodimer in CHO supernatants was measured by CE-CGE on Protein A (ProtA) purified supernatants harvested on day 14. The actual BEAT^® ^titer was obtained by multiplying the concentration measured by HPLC-PA by the fraction of heterodimer measured by CE-CGE in ProtA purified supernatants. The BEAT^® ^was produced in 3 L STR bioreactors (Mobius CellReady Bioreactor, Millipore) in fed-batch. Supernatants were typically harvested on day 14 by centrifugation and dead-end filtration. A single Protein A step was performed for purification, where two isocratic steps allowed the selective elution of the bispecific product. The thermostability of the BEAT^® ^molecule was measured by differential scanning calorimetry (DSC) in PBS.

## Results

**Figure 1:**
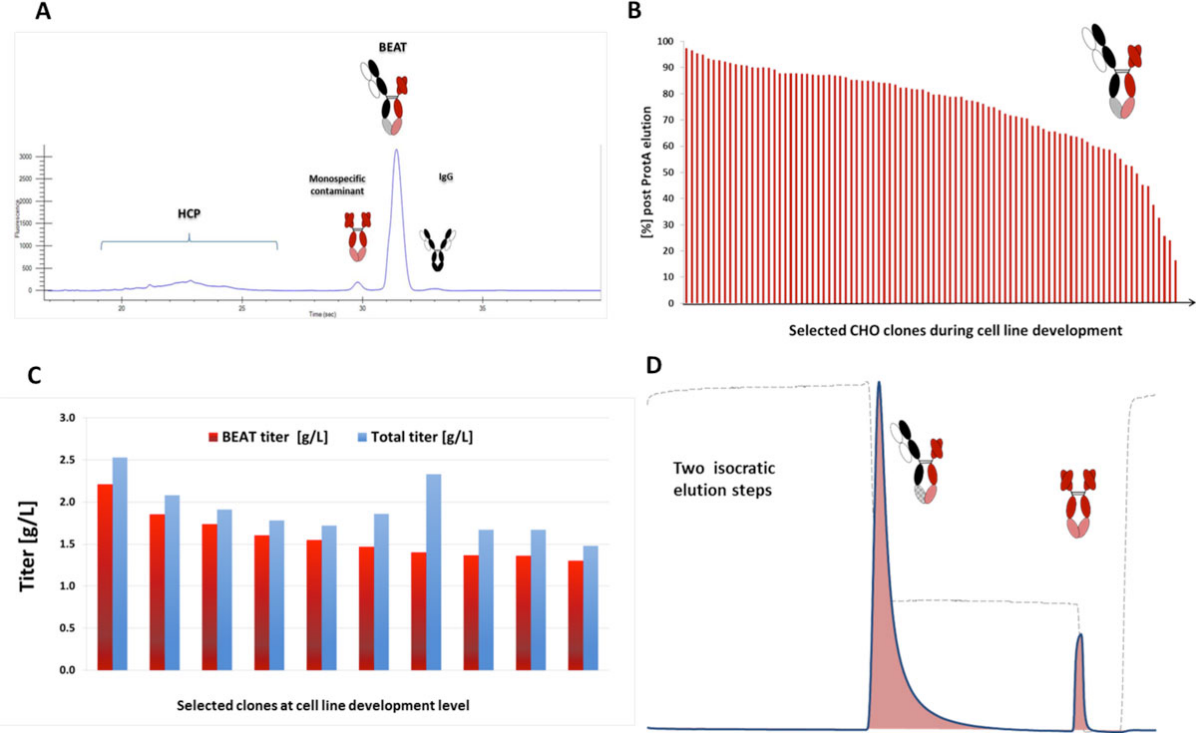
**The BEAT**^®^**bispecific platform**. In A: secretion profile of a BEAT^® ^secreting CHO clone obtained by Caliper analysis of a non-purified supernatant. B: distribution of the heterodimerization level of stable clones at cell line development level. C: BEAT^® ^expression level of 10 selected stable clones. D: BEAT^® ^purification strategy.

The BEAT^® ^bispecific molecule consists of three chains: a heavy chain (Hc), a light chain (Lc) and a Fc-scFv (see Figure 1 A). The molecule has a fully functional Fc and engages two biological targets by a Fab arm on one side and by a scFv on the other. Heterodimerization is achieved by a proprietary CH3 interface, mimicking the natural association of the T-cell surface receptors α and β between the two CH3 domains of IgG. Lc mispairing is avoided by the replacement of one Fab arm of the bispecific IgG by a scFv. In addition, the Protein A binding site in the Hc of the molecule is abrogated to facilitate the isolation of the BEAT**^®^**-antibody by affinity chromatography (discussed in the following). The DSC analysis of the BEAT^® ^indicated a good thermostability within the range of naturally occurring antibodies. The BEAT^® ^molecule is expressed in CHO cells. Figure 1 A shows a typical secretion profile obtained by Caliper Protein Analysis of a non-purified CHO supernatant after 14 days in fed-batch culture. It can be seen that the asymmetry of the BEAT^® ^format allows an easy characterization of the secretion profile of generated clones using HT analytics solely based on molecular weight. The example illustrates that a very low level of monospecific IgG is secreted and that the main secreted species is the BEAT^® ^molecule, the main monospecific contaminant being the scFv-Fc homodimer. Figure 1 B shows the distribution of the heterodimerization level of the CHO clones screened during cell line development. The median of the distribution is approx. 80 % indicating that half of the generated clones secreted > 80 % of heterodimer. The expression level of the best 10 clones selected in small scale fed-batches after cell line development can be seen in Figure 1 C. Clones secreting 1-2 g/L of BEAT**^®^**could be obtained under non-optimized fed-batch conditions. Stability studies demonstrated that selected CHO clones have a stable level of heterodimerization over long term cultivation (75 population doubling level (PDL), data not shown).

At 3 L bioreactor scale, titers of 3 g/L with 90 % of secreted heterodimer could be obtained in fed-batch with minimal feeding optimization. After harvest the molecule is purified by Protein A (ProtA). For purification purposes the BEAT^® ^was designed with a missing ProtA binding site on the Hc of the molecule. Consequently, residual monospecific IgG contaminants (harboring 2 Hc) do not bind to the ProtA column and are thus easily separated from the products of interest. In addition, the BEAT^® ^molecule and the homodimeric Fc-scFv contaminant exhibit a different affinity for Protein A as the molecules harbor one and two binding sites for ProtA, respectively. Thus, the BEAT^® ^molecule can be separated by ProtA via a two-step isocratic elution as illustrated in Figure 1 D. Applying this purification strategy for harvested bioreactor material, a level of purity of 97 % could be obtained post ProtA.

## Conclusions

This work introduces a new bispecific IgG format called the BEAT**^®^**. Glenmark's BEAT^® ^platform allows the generation of stable clones with volumetric productivity of several g/L and a high heterodimerization level (> 90 % secreted BEAT^® ^in CHO supernatants). Generated clones harbor stable product quality profiles, e.g. level of heterodimerization, over at least 75 PDL. The developed purification strategy allows a purity reaching 97 % post ProtA. The BEAT^® ^platform combines a unique CH3 interface for heterodimerization, an efficient cell line selection strategy and an industrial relevant purification process for the production of pure bispecific antibody at several g/L.
